# Whole-genome sequencing and machine learning reveal key drivers of delayed sputum conversion in rifampicin-resistant tuberculosis

**DOI:** 10.3389/fcimb.2025.1641385

**Published:** 2025-08-07

**Authors:** Qing Fang, Xiangchen Li, Yewei Lu, Junshun Gao, Yvette Wu, Yi Chen, Yang Che

**Affiliations:** ^1^ Departments of Pulmonary Medicine, The First Affiliated Hospital of Ningbo University, Ningbo, Zhejiang, China; ^2^ Jiaxing Key Laboratory of Clinical Laboratory Diagnostics and Translational Research, Affiliated Hospital of Jiaxing University, Jiaxing, Zhejiang, China; ^3^ Cosmos Wisdom Mass Spectrometry Center of Zhejiang University Medical School, Hangzhou, Zhejiang, China; ^4^ Fountain Valley School, Colorado Springs, CO, United States; ^5^ Institute of Tuberculosis Prevention and Control, Ningbo Municipal Center for Disease Control and Prevention, Ningbo, Zhejiang, China

**Keywords:** rifampicin-resistant tuberculosis, whole-genome sequencing, sputum culture conversion, machine learning, drug resistance mutations

## Abstract

Rifampicin-resistant tuberculosis (RR-TB) remains a major global health challenge, with delayed sputum culture conversion (SCC) predicting poor treatment outcomes. This study integrated whole-genome sequencing (WGS) and machine learning to identify clinical and genomic determinants of SCC failure in 150 RR-TB patients (2019–2023). Phenotypic and genotypic analysis revealed high rates of isoniazid resistance (74.0%) and *rpoB* mutations (97.3%, predominantly Ser450Leu), with 90% of strains belonging to Lineage 2 (Beijing family). While 64.7% achieved 2-month SCC, 18.0% remained culture-positive at 6 months. Univariate analysis linked 2-month SCC failure to smear positivity, resistance to isoniazid, amikacin, capreomycin, and levofloxacin, and pre-XDR-TB status, though only smear positivity (aOR=2.41, P=0.008) and levofloxacin resistance (aOR=2.83, P=0.003) persisted as independent predictors in multivariable analysis. A Random Forest model achieved robust prediction of SCC failure (AUC: 0.86 ± 0.06 at 2 months; 0.76 ± 0.10 at 6 months), identifying levofloxacin resistance (feature importance: 6.37), *embB*_p.Met306Ile (5.94), and smear positivity (5.12) as top 2-month predictors, while *katG*_p.Ser315Thr (4.85) and *gyrA*_p.Asp94Gly (3.43) dominated 6-month predictions. These findings underscore smear positivity, levofloxacin resistance, and specific resistance mutations as critical drivers of SCC failure, guiding targeted RR-TB treatment strategies.

## Introduction

Tuberculosis (TB) remains a major global health challenge, with an estimated 10 million new cases and 1.5 million deaths annually (Bagcchi, 2023). Rifampicin-resistant tuberculosis (RR-TB) is defined as TB resistant to at least rifampicin (RIF). The emergence of drug-resistant TB, particularly RR-TB, poses a significant threat to global TB control efforts. RR-TB, which includes multidrug-resistant TB (MDR-TB) and extensively drug-resistant TB (XDR-TB), is associated with higher mortality rates, prolonged treatment durations, and increased healthcare costs. In 2020, almost half a million individuals developed RR-TB, contributing to an estimated 6.9 million disability-adjusted life years (DALYs) (Menzies et al., 2023). The global burden of RR-TB underscores the urgent need for improved diagnostic and treatment strategies to address this growing public health crisis.

Sputum culture conversion (SCC), defined as the absence of *Mycobacterium tuberculosis* (MTB) growth in sputum culture, is a key indicator of treatment efficacy in TB management (Holtz et al., 2006). In RR-TB, SCC is often delayed due to drug resistance, highlighting the need to identify factors influencing conversion to optimize treatment outcomes. Delayed SCC is associated with treatment failure, relapse, and poor prognosis, underscoring its prognostic significance (Wenlu et al., 2024). Clinically, SCC is typically assessed at two critical time points: 2 and 6 months after treatment initiation. While 2-month SCC reflects early bacterial suppression, its predictive value for long-term outcomes is limited, especially in patients with comorbidities such as HIV (Kurbatova et al., 2015). In contrast, 6-month SCC serves as a reliable predictor of sustained bacterial clearance and treatment success, providing critical guidance for clinical decision-making and outcome prediction (Meyvisch et al., 2018).

Prior studies have explored demographic, clinical, and resistance-related factors influencing SCC in TB, yet research specific to RR-TB remains limited (Liu et al., 2018). The impact of genetic mutations on SCC outcomes in RR-TB is particularly underexplored. Emerging evidence suggests that specific MTB mutations, such as those in *inhA* and *katG*, are associated with increased second-line drug resistance and delayed SCC, highlighting the critical role of genetic factors in treatment response (Click et al., 2020). Whole-genome sequencing (WGS) provides a robust tool for comprehensive mutation detection and phylogenetic lineage classification, which may affect bacterial fitness and therapeutic outcomes (Meehan et al., 2019; He et al., 2020). Moreover, machine learning (ML) algorithms can effectively integrate high-dimensional clinical and genomic data to predict treatment outcomes with precision and prioritize key predictors, complementing traditional statistical approaches (Chafai et al., 2024). However, the combined application of WGS and machine learning to identify SCC determinants in RR-TB remains in its early stages, with the impact of specific resistance mutations on SCC timing still poorly understood (Kurbatova et al., 2015). Addressing these gaps is crucial for developing targeted interventions to enhance RR-TB treatment success.

This study aims to elucidate the clinical and genetic determinants of SCC in RR-TB using a WGS-based approach. We analyzed clinical data and isolates from 150 RR-TB patients diagnosed between January 2021 and September 2023, assessing the association of demographic, clinical, and microbiological characteristics with 2- and 6-month SCC outcomes. Phenotypic and genotypic resistance profiles were characterized through drug susceptibility testing and WGS, with a focus on identifying specific resistance mutations linked to SCC. By integrating clinical, genomic, and machine−learning–driven insights, this study seeks to uncover factors contributing to delayed SCC, inform precision treatment strategies, and ultimately advance RR−TB management and control.

## Materials and methods

### Study design and sample enrollment

The study was a retrospective study that included all the culture-positive patients diagnosed with RR-TB at local TB dispensaries in Ningbo, China from Jan 1, 2021, to Dec 31, 2023. Patients aged above 18 years with sputum culture-positive, pulmonary, RR-TB were assessed for eligibility, and those with consent to standardized RR-TB regimen were included. Patients were excluded if they were pregnant or infected with HIV, hepatitis B or C virus, or refused to participate. Records related to demographics, clinical and microbiology were retrieved from the national TB information management system.

### Main definitions

The response to treatment in this study was evaluated by: 2-month sputum culture conversion as a marker of early treatment response, 6-month culture conversion (previously reported to be predictive of treatment outcome). Sputum culture conversion was defined as two consecutive negative cultures of samples taken at least 30 days apart (Mirzayev et al., 2021). This definition was strictly maintained for all timepoint evaluations (2/6 months).

### Drug susceptibility test

Drug susceptibility tests of four first-line anti-TB drugs and five second-line drugs were conducted on solid media (Lowenstein-Jensen) based on WHO recommendations (Bagcchi, 2023). The drug concentrations are isoniazid (INH) 0.2 µg/ml, rifampicin (RIF) 40 µg/ml, ethambutol (EMB) 2 µg/ml, streptomycin (SM) 4 µg/ml, levofloxacin (LVX) 2 µg/ml, amikacin (AM) 30 µg/ml, capreomycin (CM) 40 µg/ml, prothionamide (PTO) 40 µg/ml, and para-aminosalicylic acid (PAS) 1 µg/ml. H37RV strains were used as a reference for quality control.

### WGS and bioinformatics analysis

MTB culture products were inactivated, and genomic DNA was isolated using a bacterial DNA extraction kit (QIAGEN Inc., Dusseldorf, Germany), according to the manufacturer’s instructions. The isolated and purified DNA products were transported via a cold chain to a sequencing facility. The purified genomic DNA was quantified using a TBS-380 fluorometer (Turner BioSystems Inc., Sunnyvale, CA, USA) to ensure that the DNA met the quality requirements for library preparation, sequencing, and detection. At least 1 μg of genomic DNA per sample was used as the input material for DNA sample preparation. The DNA samples were treated and fragmented to a size of ~400 bp. Sequencing libraries were generated using the NEXTflex™ Rapid DNA-Seq Kit. The prepared library was multiplexed and loaded on Illumina NovaSeq 6000 PE150 system (San Diego, CA92122, USA). Sequencing was carried out using a 2×150 paired-end (PE) configuration. Raw sequencing data were processed using fastp (v0.20.1) to remove adapter sequences and filter out low-quality bases (Chen et al., 2018). High-quality sequence data were then input into Kraken v2 for species identification, and samples identified as other species or with an MTB proportion below 90% were rejected as contaminated samples (Wood et al., 2019). Finally, the sequencing data from the remaining samples were aligned to the H37Rv reference genome (NC_000962.3) using BWA (v0.7.17) (Li, 2013). Samples with an average sequencing depth > 20× and average genome coverage >95% were selected for subsequent data analysis. The SAMtools/BCFtools suite was used for calling fixed (frequency ≥90%) SNPs at loci where the alternate alleles were supported by at least five reads (including both forward and reverse reads) (Danecek et al., 2021).

### Resistance mutation analysis

Clean sequencing data were input into the local version of TB-Profiler (v6.5.0) with its strict implementation of the V2 catalog (database name: who) to identify the genotype of resistance-associated mutations and detect the resistance profile of 15 anti-TB drugs, including Amikacin (AM), Bedaquiline (BDQ), Capreomycin (CM), Clofazimine (CFZ), Delamanid (DLM), Ethambutol (EMB), Ethionamide (ETO), Isoniazid (INH), Kanamycin, Levofloxacin (LVX), Linezolid (LZD), Moxifloxacin (MFX), Pyrazinamide (PZA), Rifampicin (RIF), Streptomycin (SM). Mutations with a frequency of less than 10% were excluded. WGS-based drug susceptibility testing (DST) results were determined by assessing the presence or absence of resistance-associated mutations in a WHO-recommended database, which classified mutations into Tier 1 (those most likely to confer resistance) and Tier 2 (genes with a reasonable pretest probability of resistance) (Walker et al., 2022). Hetero-resistance was defined based on the frequency of resistant alleles in the sequence reads <99% in this study.

### Phylogeny construction

The fixed SNPs, excluding those in SNPs in PE/PPE areas, insertion elements, repetitive regions, and drug resistance-associated genes, were combined into a concatenated alignment (Luo et al., 2014). Maximum-likelihood (ML) phylogenetic trees were inferred from the concatenated alignment using IQ-Tree v2 (Nguyen et al., 2015). The best-scoring ML tree were rooted using *M. canettii* (RefSeq: NC_015848.1) as the outgroup and visualized with the Interactive Tree of Life (iTOL) (Letunic and Bork, 2021).

### Association analysis

Statistical analyses were performed in R package gtsummary (Sjoberg et al., 2021). To compare multiple categorical variables, binary logistic regression was employed where appropriate, with results reported as odds ratios (ORs) and 95% confidence intervals (CIs). Variables showing a significant association (*P* < 0.05) in univariate logistic regression were included in the multivariate analysis. Forward stepwise logistic regression was then performed to identify whether these statistically significant covariates were independently associated with SCC.

### Machine learning analysis

A Random Forest (RF) model was implemented in R v4.0 with a fixed seed of 27 to identify genetic mutations and clinical factors associated with delayed SCC at 2 and 6 months in RR-TB. RF was chosen for its ability to handle high-dimensional, nonlinear data, robustness to overfitting, and capacity to rank feature importance, making it well-suited for integrating clinical and genomic predictors compared to other models like logistic regression or support vector machines. Mutations in DR related genes and clinical metadata from 150 isolates were merged. Clinical and phenotypic DST features were encoded, and mutations imputed with 0. Mutations in ≥5% of samples were selected. Class imbalance was addressed using SMOTE (DMwR package) (Kumari et al., 2020). The RF model was trained using the caret package’s train function (randomForest method, 100 trees) with an 80/20 train-test split and 5-fold cross-validation (ROC-AUC). Hyperparameter tuning was performed with tuneLength = 3, optimizing the number of variables sampled at each split (mtry). Feature importance in the RF models was assessed using mean decrease in accuracy (MDA), with standard deviations estimated from 100 bootstrap iterations to quantify variability. Test-set AUC and its standard deviation were estimated using 100 bootstrap iterations. Feature importance and ROC curves were visualized using ggplot2 (Villanueva and Chen, 2019).

## Results

### Study population and characteristics of RR-TB patients

**Figure 1:**
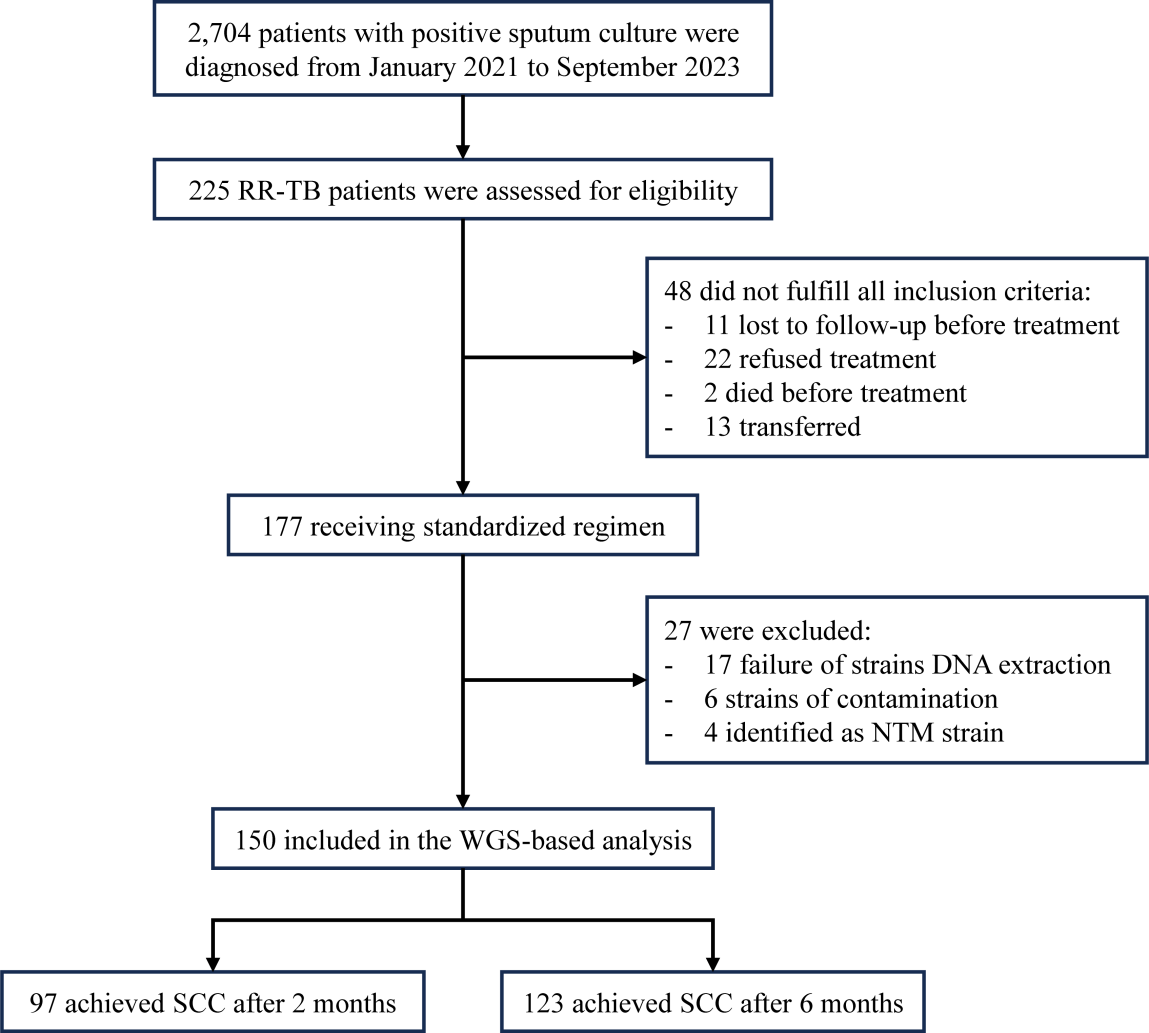
Flowchart of this study.

Between January 2021 and September 2023, a total of 2,704 patients were diagnosed with sputum culture-positive tuberculosis, among whom 225 were identified as having RR-TB. From 177 initially enrolled patients, 48 were excluded: 11 lost to follow-up pre-treatment, 22 declined treatment, 2 died pre-enrollment, and 13 transferred. During WGS processing, 27 additional cases were excluded: 17 from culture failure, six due to contamination (<90% MTBC sequences), and four confirmed as NTM infections (three *M. intracellulare*, one *M. abscessus*) by Kraken2 analysis. Ultimately, 150 patients with successful WGS results were included in the final analysis (**Figure 1**). Among the 150 RR-TB patients, 97 patients (64.7%) achieved SCC after 2 months of treatment. By 6 months of treatment, the number of patients who successfully achieved SCC increased to 123 (82.0%).

As shown in **Table 1**, the mean age of patients was 45 years, and 117 (78.0%) were male. A total of 72 patients (48.0%) were smear-positive. Retreatment cases accounted for 44.0% ( n=66 ). Underlying health conditions were reported in 62 patients (42.7%), with diabetes (53.2%, 33/62) and hypertension (35.5%, 22/62) being the most common. Additionally, 24 patients were smokers, and 21 reported alcohol consumption. Migrant individuals comprised 46.0% ( n=60 ) of the cohort. Regarding occupation, the largest groups were workers (32.7%, n=49 ), unemployed individuals (29.3%, n=44 ), and farmers (20.0%, n=30 ).

**Table 1:** Demographic and clinical characteristics of patients with RR-TB in this study.

Characteristics	Number
Age, mean ± SD	45 ± 16.1
Male sex Smear-positive	114 (76.0%) 72 (48.0%)
Medical history	62 (41.3%)
Smoking	33 (22.0%)
Drunk	21 (14.0%)
Retreatment	66 (44.0%)
Migrant	69 (46.0%)
Occupation	
Farmer	30 (20.0%)
Unemployed	44 (29.0%)
Worker	50 (33.0%)
Other	26 (17.0%)
Phenotypic drug resistance	
INH	111.0 (74.0%)
SM	93.0 (62.0%)
EMB	50.0 (33.3%)
AM	13.0 (8.7%)
CM	10.0 (6.7%)
LVX	54.0 (36.0%)
PAS	25.0 (16.7%)
PTO	23.0 (15.3%)

### Phenotypic and genotypic profiles of RR-TB isolates

Excluding RIF, phenotypic DST revealed that resistance rates for the remaining eight anti-tuberculosis drugs ranged from highest to lowest as follows (**Table 1**): isoniazid (INH), 74.0% (111/150); streptomycin (SM), 62.0% (93/150); levofloxacin (LVX), 36.0% (54/150); ethambutol (EMB), 33.3% (50/150); para-aminosalicylic acid (PAS), 16.7% (25/150); prothionamide (PTO), 15.3% (23/150); amikacin (AM), 8.7% (13/150); and capreomycin (CM), 6.7% (10/150).

A total of 133 mutations were identified by WGS across 19 antibiotic resistance genes related to RIF, INH, EMB, PZA, ETO, SM, PAS, aminoglycosides, and fluoroquinolones resistance (**Supplementary Table S1**). Genomic analysis revealed RIF-resistance-associated mutations in 98.7% (148/150) of isolates, predominantly in *rpoB* (146 strains), with rare *rpoC* mutations (2 strains). The most frequent *rpoB* mutation was Ser450Leu, accounting for 58.2% (85/146) of cases, followed by Leu452Pro (12.3%, 18/146), with other low-frequency mutations (e.g., Leu430Pro, Asp435Ala). Additionally, two strains carried *rpoC* mutations (Ile435Thr, Phe452Ser). Beyond RIF, mutations conferring resistance to other drugs were detected, including *embB*_p.Met306Ile (EMB), *katG*_p.Ser315Thr (INH), *gyrA*_p.Asp94Gly (fluoroquinolones), and *rrs*_n.1401A>G (aminoglycosides), reflecting the multidrug-resistant nature of the cohort. Based on WGS results and WHO’s updated definitions, the 150 RR-TB strains were further categorized into 65 MDR-TB, 51 pre-XDR-TB, and 34 RR-TB cases. These findings highlight the genetic diversity of resistance in RR-TB and underscore the utility of WGS in identifying critical mutations driving treatment challenges.

**Figure 2:**
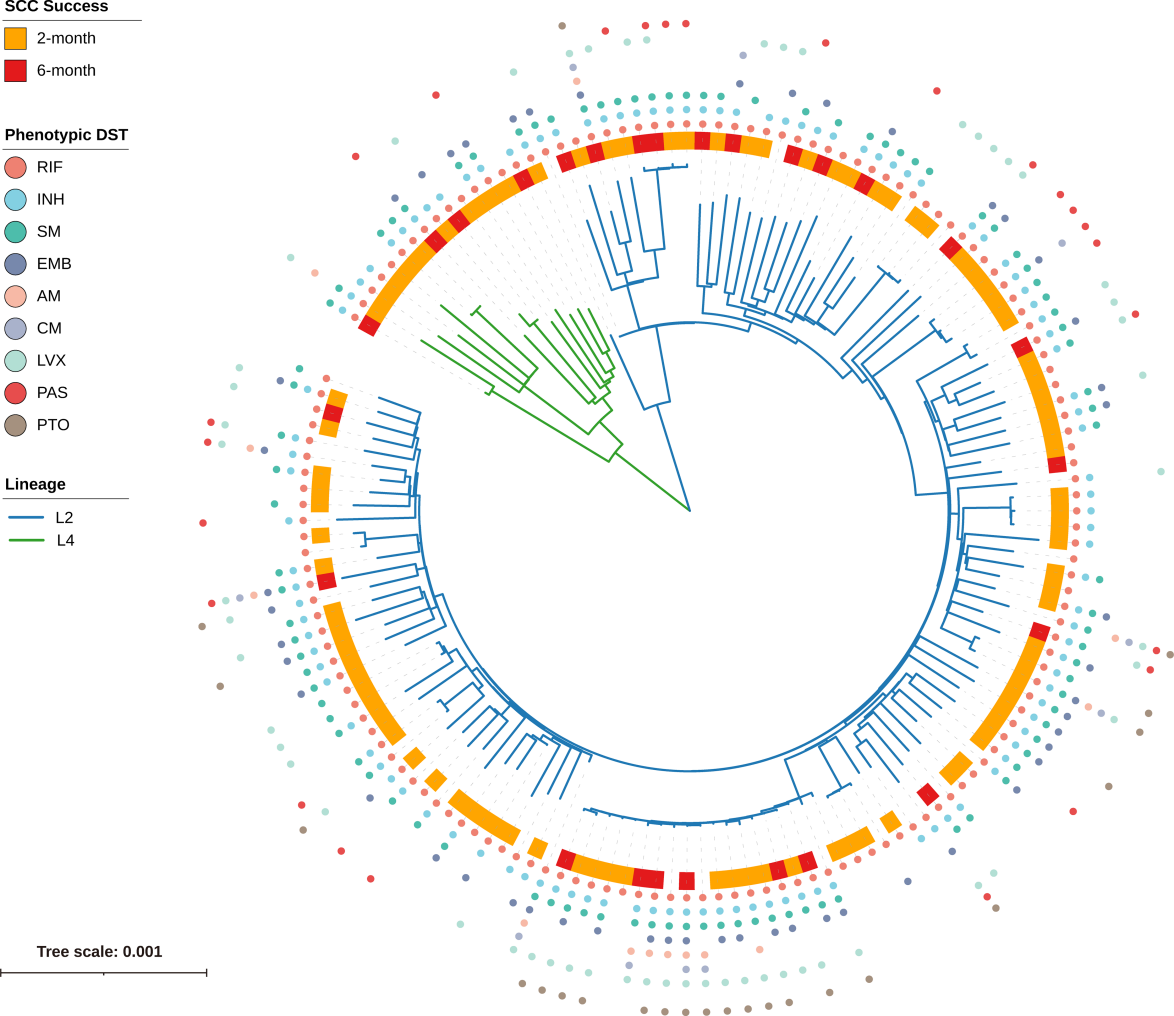
Phylogenetic tree and phenotypic DST profile of 150 RR-TB strains. The maximum-likelihood phylogenetic tree is based on concatenated SNP loci, with branch lengths representing the number of nucleotide substitutions per site. Branch colors represent lineages: blue for L2 and green for L4. The outer ring indicates sputum culture conversion (SCC) status: orange for SCC achieved at 2 months, red for SCC at 6 months, and white for no SCC by 6 months. The outermost colored dots denote phenotypic resistance to nine anti-TB drugs (RIF, INH, SM, EMB, AM, CM, LVX, PAS, PTO), with each drug assigned a unique color as indicated in the legend.

To elucidate the evolutionary relationships of the 150 RR-TB strains, we constructed a ML phylogenetic tree based on concatenated sequences from non-redundant SNP loci (**Figure 2**). Genotyping analysis revealed that 90.0% (135/150) of the strains were classified into lineage 2 (L2), with the majority falling under the L2.2.1 ( n=125 ) and L2.2.2 ( n=9 ) sublineages (Beijing family), accounting for a total of 134 strains. The remaining 10.0% (15/150) belonged to lineage 4 (L4), comprising sublineages L4.4 ( n=7 ), L4.5 ( n=6 ), and L4.2 ( n=2 ), respectively.

### Demographic and clinical factors influencing the SCC in RR-TB

Univariate analysis of 150 RR-TB patients identified factors associated with the SCC failure at 2- and 6-month post-treatment initiation (**Table 2**). At 2-month, SCC failure showed significant associations ( OR=3.15, 95% CI: 1.58−6.46, P=0.001 ) with smear-positive status, phenotypic resistance to (INH OR=3.24, 95% CI: 1.38−8.55, P=0.011 ), AM ( OR=7.29, 95% CI: 2.11−33.7, P=0.004 ), CM ( OR=8.44, 95% CI: 2.02−57.5, P=0.009 ), LVX ( OR=4.53, 95% CI: 2.23−9.45, P<0.001 ), and genotypic pre-XDR-TB ( OR=4.70, 95% CI: 1.80−13.5, P=0.002 ). However, by 6-month, only smear-positive status and LVX resistance remained significantly associated with delayed conversion ( OR=3.25, 95% CI: 1.39−7.86, P=0.007 ), indicating that other resistance profiles became less influential over time.

**Table 2:** Risk factors for SCC failure at 2 and 6 months: univariable and multivariable regression analysis.

	Univariable (2-month)				Multivariable (2-month)			Univariable (6-month)			Multivariable (2-month)		
	N	OR*^1^*	95% CI*^1^*	p-value	AOR*^1^*	95% CI*^1^*	p-value	OR*^1^*	95% CI*^1^*	p-value	AOR*^1^*	95% CI*^1^*	p-value
Sex	150												
female		—	—					—	—				
male		1.33	0.60, 3.06	0.5				2.93	0.94, 12.9	0.10			
Age	150												
<60 y		—	—					—	—				
>60 y		1.38	0.55, 3.34	0.5				2.18	0.76, 5.80	0.13			
Sputum smear	150												
Negative		—	—		—	—		—	—		—	—	
Positive		3.15	1.58, 6.46	**0.001**	3.32	1.54, 7.44	**0.003**	3.14	1.31, 8.12	**0.013**	2.90	1.19, 7.61	**0.023**
Smoking	150												
No		—	—					—	—				
Yes		1.06	0.46, 2.34	0.9				2.06	0.80, 5.08	0.12			
Drunk	150												
No		—	—					—	—				
Yes		1.15	0.43, 2.94	0.8				2.06	0.67, 5.73	0.2			
Registered residence	150												
Local		—	—					—	—				
Migrant		1.36	0.69, 2.67	0.4				0.93	0.39, 2.14	0.9			
Occupation	150												
unemployed		—	—					—	—				
worker		0.56	0.23, 1.32	0.2				1.01	0.33, 3.13	>0.9			
farmer		0.96	0.37, 2.48	>0.9				2.64	0.88, 8.32	0.086			
other		0.76	0.27, 2.07	0.6				0.44	0.06, 2.01	0.3			
TB history	150												
No		—	—					—	—				
Yes		1.96	1.00, 3.89	0.052				1.76	0.76, 4.15	0.2			
Treatment history	150												
Initial treatment		—	—					—	—				
retreatment		1.96	1.00, 3.89	0.052				1.76	0.76, 4.15	0.2			
INH	150												
S		—	—		—	—		—	—				
R		3.24	1.38, 8.55	**0.011**	4.02	0.89, 23.4	0.088	1.68	0.63, 5.33	0.3			
SM	150												
S		—	—					—	—				
R		1.49	0.74, 3.05	0.3				0.72	0.31, 1.70	0.4			
EMB	150												
S		—	—					—	—				
R		1.75	0.87, 3.53	0.12				1.22	0.50, 2.87	0.7			
AM	150												
S		—	—		—	—		—	—				
R		7.29	2.11, 33.7	**0.004**	1.43	0.25, 9.37	0.7	3.27	0.92, 10.8	0.055			
CM	150												
S		—	—		—	—		—	—				
R		8.44	2.02, 57.5	**0.009**	3.64	0.51, 31.6	0.2	2.07	0.42, 8.05	0.3			
LVX	150												
S		—	—		—	—		—	—		—	—	
R		4.53	2.23, 9.45	**<0.001**	6.09	1.31, 35.0	**0.026**	3.25	1.39, 7.86	**0.007**	3.01	1.27, 7.40	**0.013**
PAS	150												
S		—	—					—	—				
R		2.30	0.96, 5.56	0.060				2.04	0.72, 5.38	0.2			
PTO	150												
S		—	—					—	—				
R		1.86	0.75, 4.58	0.2				2.34	0.81, 6.27	0.10			
Lineage	150												
L2		—	—					—	—				
L4		0.64	0.17, 1.98	0.5				0.00		>0.9			
Genotypic DR type	150												
RR-TB		—	—		—	—		—	—				
MDR-TB		1.48	0.56, 4.21	0.4	0.54	0.09, 2.95	0.5	0.56	0.17, 1.90	0.3			
Pre-XDR-TB		4.70	1.80, 13.5	**0.002**	0.29	0.03, 2.20	0.3	1.77	0.62, 5.52	0.3			

^1^OR, Odds Ratio; CI, Confidence Interval; AOR, Adjusted Odds Ratio.

Significant results (P < 0.05) are shown in bold.

To identify independent predictors of delayed SCC, we constructed multivariable logistic regression models. For 2-month conversion failure, the model was adjusted for smear-positive status, phenotypic resistance to INH, AM, CM and LVX, as well as genotypic pre-XDR-TB. For 6-month failure, adjustments included smear-positive status and LVX resistance. After adjustment, smear positivity ( aOR=3.32, 95% CI:1.54−7.44, P=0.003 ) and LVX resistance ( aOR=6.09, 95% CI:1.31−35.0, P=0.026 ) were significantly associated with 2-month SCC failure. Other factors in the 2-month model, including INH, AM, CM resistance, and genotypic pre-XDR-TB were not statistically significant. For 6-month SCC failure, smear positivity ( aOR=2.90, 95% CI:1.19−7.61, P=0.023 ) and LVX resistance ( aOR=3.01, 95% CI:1.27−7.40, P=0.013 ) remained significant predictors. The strong association of smear positivity and LVX resistance with SCC failure suggested that higher bacterial burden and fluoroquinolone resistance significantly impede early and sustained treatment response.

### ML analysis of delayed SCC

**Figure 3:**
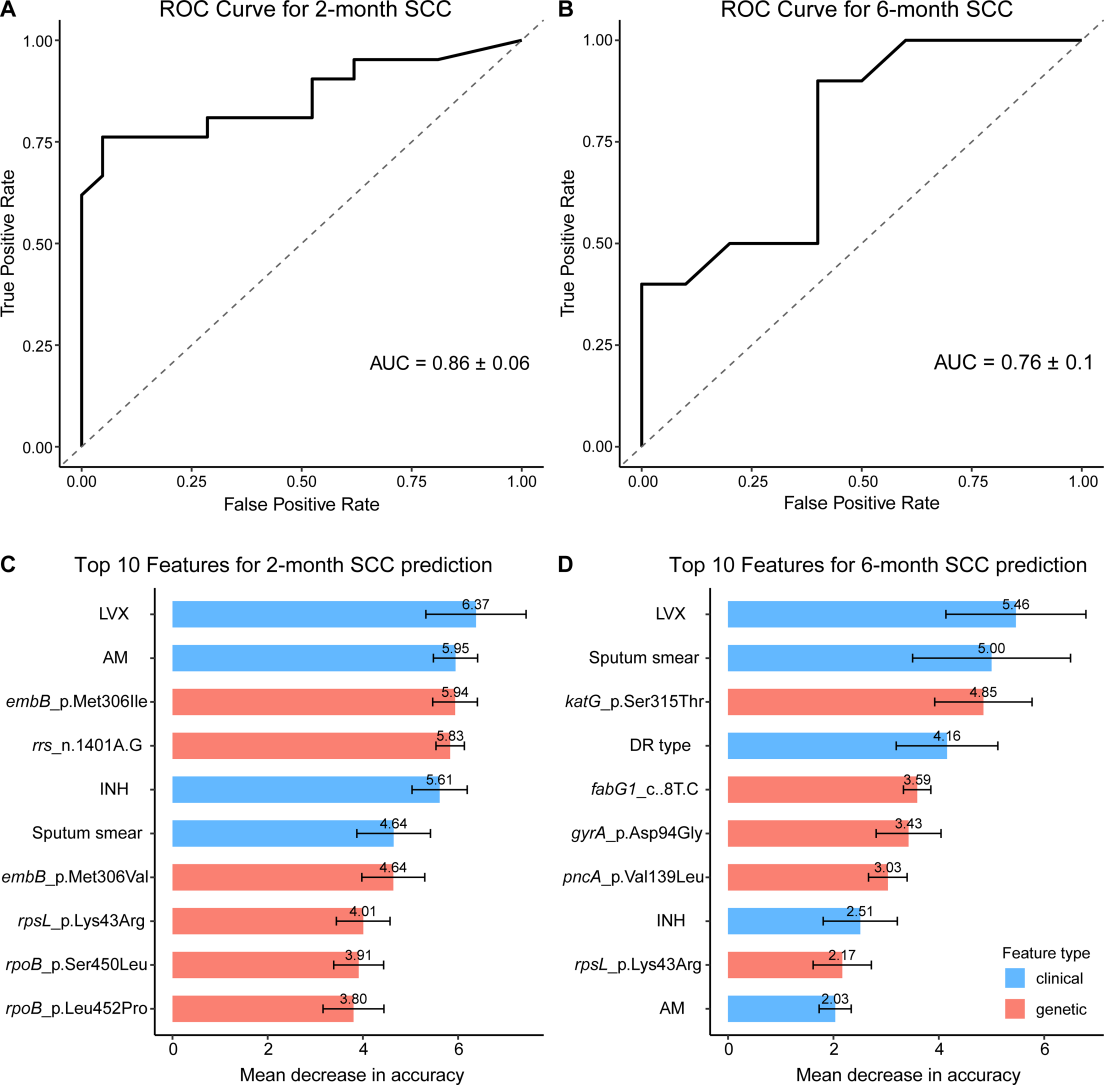
Random forest model performance and feature importance for predicting SCC Failure in RR-TB Patients. **(A, B)** Receiver Operating Characteristic (ROC) curves for predicting sputum culture conversion (SCC) failure at 2 months **(A)** and 6 months **(B)**, based on 5-fold cross-validation. The dashed line indicates random guessing (AUC = 0.5). **(C, D)** Bar plots of the top 10 predictors of SCC failure at 2 months **(C)** and 6 months **(D)**, measured as Mean Decrease in Accuracy (MDA), with higher values indicating greater predictive importance. Clinical features (e.g., smear positivity) are shown in blue, and genetic mutations (e.g., katG_p.Ser315Thr) in red. Importance values are displayed above each bar, with error bars representing standard deviations from 100 bootstrap iterations.

The RF model demonstrated robust performance in predicting SCC failure at both 2 and 6 months among patients with RR-TB, yielding ROC-AUC values of 0.86 ± 0.06 and 0.763 ± 0.103, respectively, based on 5-fold cross-validation (**Figures 3A, B**). Feature importance analysis measured as MDA, highlighted key determinants of delayed SCC with variability estimated from 100 bootstrap iterations (**Figures 3C, D**). For the 2-month endpoint, the most influential predictors included LVX resistance ( MDA =6.37 ± 1.05 ), AM resistance ( MDA=5.95 ± 0.46 ), and the *embB*_p.Met306Ile mutation (EMB resistance, MDA=5.94 ± 0.47 ), followed by *rrs*_n.1401A>G (AM resistance, MDA=5.83 ± 0.30 ) and INH resistance ( MDA=5.61 ± 0.58 ). At 6 months, the top contributors were LVX resistance ( MDA=5.46 ± 1.33 ), baseline sputum smear positivity ( MDA=5.00 ± 1.50 ), and the *katG*_p.Ser315Thr mutation (INH resistance, MDA=4.85 ± 0.92 ), with genotypic DR type ( MDA=4.16 ± 0.96 ) and *gyrA*_p.Asp94Gly (fluoroquinolone resistance, MDA=3.43 ± 0.62 ) also playing significant roles. Notably, *katG*_p.Ser315Thr and *gyrA*_p.Asp94Gly emerged as stronger predictors at the 6-month mark, underscoring their association with prolonged treatment failure due to INH and fluoroquinolone resistance, respectively.

## Discussion

This study provides a comprehensive analysis of clinical and genomic determinants of delayed SCC in RR-TB, combining WGS and ML to unravel critical predictors of treatment failure. Our findings underscore the persistent challenge of delayed SCC in RR-TB, with 35.3% of patients failing to achieve conversion at 2 months and 18.0% remaining culture-positive at 6 months. These rates align with prior reports of poor SCC outcomes in RR-TB cohorts, particularly in high-burden settings, but extend existing knowledge by dissecting the interplay of phenotypic resistance, genetic mutations, and bacterial lineage dynamics.

The association of smear positivity with delayed SCC at both time points reinforces its role as a biomarker of high bacterial burden and poor treatment response. This aligns with studies by Kurbatova et al, who demonstrated that smear grade correlates with prolonged culture positivity in MDR-TB (Kurbatova et al., 2015). However, our work uniquely identifies LVX resistance as a persistent independent predictor of SCC failure, even after adjusting for confounding resistance profiles. This finding is critical in light of global shifts toward shorter, fluoroquinolone-intensive regimens for RR-TB (Pranger et al., 2019). Our results corroborate earlier evidence that fluoroquinolone resistance undermines bactericidal activity, but further highlight that its impact persists beyond the early treatment phase, likely due to compensatory mutations in gyrase genes (e.g., *gyrA*_p.Asp94Gly) that enhance fitness under drug pressure (Pantel et al., 2016; Diriba et al., 2022).

The diminishing significance of aminoglycoside resistance (AM/CM) in multivariable models at 6 months contrasts with its prominence at 2 months. This temporal divergence may reflect the delayed sterilizing effects of second-line injectables, which are often prioritized in early-phase regimens but phased out later (Conradie et al., 2022). Similarly, the loss of association between pre-XDR-TB and SCC failure in adjusted analyses suggests that resistance complexity alone may not drive outcomes if core drugs (e.g., bedaquiline, linezolid) retain efficacy—a hypothesis supported by recent trials (Trevisi et al., 2023).

By integrating WGS with ML, this study advances beyond traditional regression approaches, capturing nonlinear interactions among predictors. The Random Forest model’s high accuracy (ROC-AUC: 0.86 at 2 months) outperforms prior SCC prediction tools reliant on clinical variables alone (Kurbatova et al., 2015). Importantly, our ML framework prioritized mutations (e.g., *rrs*_n.1401A>G for aminoglycoside resistance) that are rarely assessed in phenotypic DST but critically influence SCC. This supports the WHO’s push for expanded genetic DST in RR-TB management (World Health Organization, 2021).

Notably, ML-driven feature importance analysis uncovered mutation-specific temporal effects. While *embB*_p.Met306Ile dominated 2-month SCC failure—potentially by compromising EMB’s role in early bacterial suppression—*katG*_p.Ser315Thr and *gyrA*_p.Asp94Gly emerged as stronger predictors at 6 months. This aligns with mechanistic studies showing that *katG* mutations confer enduring isoniazid resistance via catalase-peroxidase inactivation (Ando et al., 2010), while *gyrA* mutations stabilize DNA gyrase under prolonged fluoroquinolone exposure (Aldred et al., 2014). Such findings underscore the need for dynamic, mutation-adjusted treatment strategies.

Phylogenetic analysis revealed that 90.0% of the isolates belonged to the L2 Beijing family and 10.0% to L4, consistent with the epidemiological profile of RR-TB in Eastern China (Yang et al., 2017). While no significant lineage-specific SCC effects were detected in our analysis, their potential impact cannot be entirely ruled out (Liu et al., 2020). Previous studies suggest that Beijing strains may exhibit higher drug resistance acquisition due to increased fitness and compensatory mutations, such as those in *rpoC* and *katG*, which mitigate the fitness cost of rifampicin resistance (Nguyen et al., 2025). Additionally, our ML analysis prioritized specific mutations (e.g., *katG*_p.Ser315Thr, *gyrA*_p.Asp94Gly) over lineage itself, suggesting that mutation-driven resistance profiles may outweigh lineage-specific effects in determining SCC outcomes. Future studies with more balanced lineage representation are needed to quantify the direct impact of lineages on SCC and explore interactions with resistance mutations.​

These findings have immediate clinical implications. First, rapid detection of LVX resistance through genotypic assays should guide regimen selection, avoiding empiric fluoroquinolone use in high-resistance settings (Pillay et al., 2022). Second, smear-positive patients require intensified monitoring, with early escalation to novel agents (e.g., pretomanid) if SCC is delayed (Dooley et al., 2023). Third, lineage-specific therapeutic approaches may be warranted, particularly for L2 strains harboring *katG*/*embB* mutations that may benefit from adjunctive therapies (Gupta et al., 2020).

Study limitations include potential selection bias from excluding 75 of 225 RR-TB cases (33%) due to objective reasons (48 cases, e.g., loss to follow-up, refusal, death, transfer) or laboratory issues (27 cases, e.g., failed isolate recovery, contamination, nontuberculous mycobacteria), which may bias delayed SCC findings by altering the impact of predictors like smear positivity or resistance mutations. Due to incomplete records in the national TB information management system, socioeconomic factors (e.g., income, education, housing), treatment adherence, and prior anti-tuberculosis drug exposure were excluded, potentially biasing our models toward microbiological and genetic predictors by overestimating the impact of resistance mutations and smear positivity on SCC failure, particularly in retreatment or socially vulnerable patients (Nidoi et al., 2021). It should be noted that China’s free treatment policy and adjustment for proxy variables (migrant status/occupation) likely mitigated this bias (Long et al., 2021). The predominance of L2 strains may limit generalizability to settings where other lineages dominate. Future studies should integrate standardized socioeconomic assessments and objective adherence monitoring.

In conclusion, our study demonstrates that delayed sputum culture conversion in RR-TB is driven by a complex interplay of clinical, microbiological, and genetic factors, with smear positivity and LVX resistance emerging as persistent predictors of poor treatment response. By integrating WGS with ML, we identified key resistance mutations (e.g., *katG*_p.Ser315Thr, *gyrA*_p.Asp94Gly) that exert time-dependent effects on SCC outcomes, providing novel insights into the dynamic nature of drug resistance in RR-TB. These findings underscore the critical need for rapid genotypic DST to guide personalized regimen selection, particularly in high-burden settings where fluoroquinolone resistance and Beijing lineage strains predominate. Our results support the incorporation of WGS-based resistance profiling into clinical decision-making to optimize RR-TB management, while highlighting the potential of machine learning to improve outcome prediction. Future studies should explore the functional mechanisms underlying these mutation-specific effects and validate our model in diverse epidemiological settings to advance precision medicine approaches for drug-resistant tuberculosis.

## Data Availability

The datasets presented in this study can be found in online repositories. The names of the repository/repositories and accession number(s) can be found in the article/**Supplementary Material**.

## Supplementary material

Supplementary Table 1 Frequency of drug resistance mutations in 150 RR-TB strains among successful and failed cases of 2-month SCC and 6-month SCC.
